# Early screening of autism spectrum disorder in general and pediatric practices, nurseries and early child care centers: Kitcat french study protocol using a two-stage procedure

**DOI:** 10.1192/j.eurpsy.2021.420

**Published:** 2021-08-13

**Authors:** S. Marignier, N. Touil, F. Subtil, M. El Jani, S. Mardirossian, S. Gaillard, B. Kassai, S. Sonie

**Affiliations:** 1 Centre De Ressources Autisme Rhône Alpes, CH LE VINATIER, BRON, France; 2 Pôle De Santé Publique, Service De Biostatistiques, HOSPICES CIVILS DE LYON, BRON, France; 3 Epicime-cic 1407 De Lyon, HOSPICES CIVILS DE LYON, BRON, France

**Keywords:** questionnaires (M-CHAT-R/F™ + CSBS DP™-ITC), autism spectrum disorder, early screening, Neurodevelopmental disorders

## Abstract

**Introduction:**

Early screening of children at-risk to develop Autism Spectrum Disorder (ASD) needs to be improved to propose early interventions. This detection should allow diagnosis of ASD before the age of 3. An early screening performed at the general practitioner of the family should facilitate accessibility to diagnosis and a better collaboration between professionals.

**Objectives:**

Our primary objective is to estimate the positive predictive value of an early detection kit composed of 2 questionnaires (First screening: M-CHAT-R/F™ + CSBS DP™-ITC) and a confirmation of the detection with a phone call by a neuropsychologist. Patients with confirmed positive M-CHAT-R/F™ and/or CSBS DP™-ITC scores are referred to a level 2 team for pre-diagnosis and diagnosis assessment.

**Methods:**

The KitCAT study is a cohort study of 1,700 children aged 16 to 24 months seen in routine care in general or pediatric practices, or in nurseries and child care centers.

**Results:**

Seven hundred and five children have already been enrolled in the study. Twenty nine patients, ie 4.1%, (with a confirmed positive M-CHAT-R/F™ and/or CSBS DP™-ITC scores) were referred to a level 2 team where a pre-diagnosis assessment was conducted by using the following test: ADI-R, ADOS 2, BLR, WPPSI-IV and Vineland II. The diagnosis of ASD (using the same test than the pre-diagnosis) was confirmed for the first two patients aged of 3.

**Conclusions:**

The preliminary results confirm that the use of 2 questionnaires may optimize the reliability of the screening. A thousand children are still needed for the final analysis and further results are expected.

**Disclosure:**

No significant relationships.

